# Strengthening capacity for community and public engagement (CPE): a mixed-methods evaluation of the ‘DELTAS Africa CPE seed fund’ pilot

**DOI:** 10.12688/wellcomeopenres.17665.1

**Published:** 2022-03-17

**Authors:** Leah Mwangi, Lillian Mutengu, Evelyn Gitau, Imelda Bates, Justin Pulford

**Affiliations:** 1 African Population and Health Research Centre, Nairobi, Kenya; 2 Science for Africa Foundation, Nairobi, Kenya; 3 Centre for Capacity Research, Liverpool School of Tropical Medicine, Liverpool, L3 5QA, UK

**Keywords:** public engagement, capacity strengthening, Africa, early career researchers, evaluation

## Abstract

**Background: **The ‘DELTAS Africa CPE seed fund’ was a pilot scheme designed to strengthen capacity in community and public engagement (CPE) via a ‘learn by doing’ approach.   The scheme supported a total of 25 early career researchers and research support staff belonging to the DELTAS Africa network to design and implement a variety of CPE projects between August 2019 and February 2021.  We examine recipient experiences of the DELTAS Africa CPE seed fund initiative, changes in their CPE attitudes, knowledge and proficiency and their CPE practice and/or practice intentions post-award.

**Methods:** A mixed-methods process and performance evaluation drawing on three data sources: An anonymous, online knowledge, attitude and practice survey completed by CPE seed fund awardees pre- and post-project implementation (N=23); semi-structured interviews completed with a sub-sample of awardees and programme implementors (N=9); and ‘end-of-project’ reports completed by all seed fund awardees (N=25).

**Results:** All awardees described their seed fund experience in positive terms, despite invariably finding it more challenging than originally anticipated.  The combined survey, interview and end of project report data all uniformly revealed improvement in awardees’ self-reported CPE knowledge, attitudes and proficiency by completion of their respective projects.   Commitment to continued CPE activity post-award was evident in the survey data and all interviewees were adamant that they would integrate CPE within their respective research work going forward.

**Conclusion: **The DELTAS Africa CPE seed fund appeared to work successfully as a CPE capacity strengthening platform and as a vehicle for fostering longer-term interest in CPE activities.

## Introduction

Engagement with research communities and/or the wider public, referred to as community and public engagement (CPE) in this paper, is recognized as a critical element of global health research ^1, 2^. Community and public engagement is a dynamic concept, constantly evolving, with no single or simple definition. However, in general, CPE refers to two-way interactions between researchers/academics and non-academic communities intended to provide opportunities for mutual learning and benefit and describes a broad spectrum of approaches and activities based on the context, type of research, target population and intended outcomes ^1– 4^.

Community and public engagement is an under-developed field within science and academia. It remains largely unclear which types of CPE activity are most effective in which context ^2, 5^ even though evaluation and evidence-building have long been advocated ^6^. In addition, poor engagement of non-academic communities by researchers continues to be widespread ^7, 8^. This is often attributed to funding and time constraints, although researchers’ limited understanding of, and experience with CPE as a field of practice, and their lack of consideration to engage with non-specialist publics as a priority may also play a part ^7– 9^. 

Competency in CPE is a recognised characteristic of researcher excellence ^10^, yet CPE is not routinely taught in postgraduate research training. Rather, CPE competency is typically acquired during a researchers’ career through practical exposure and continued professional development ^8^. Few CPE capacity strengthening programmes have been described in the published literature. This is especially true in an African context, despite: the wide range of CPE activities that take place across the continent ^2, 11, 12^, a recognised need for CPE-capacity strengthening among African researchers ^3^, and the unintended consequences that can result when CPE is poorly practiced ^13^.

We present findings from a mixed-methods evaluation of a pilot CPE capacity strengthening programme; the ‘DELTAS Africa CPE seed fund’. This seed fund was designed to promote CPE capacity strengthening via a supported ‘learn by doing’ approach and was targeted towards early career researchers belonging to any one of 11 African-led research consortia under the DELTAS Africa Programme. Drawing on data obtained from pre-/post-implementation surveys, semi-structured interviews and document review, we examine: recipient experiences of the DELTAS Africa CPE seed fund initiative; changes in recipients’ CPE attitudes, knowledge and proficiency; and recipients’ CPE practice and/or practice intentions post-award. It is anticipated the evaluation findings will inform future iterations of the seed fund scheme as well extend current understanding of good practice in CPE capacity strengthening approaches targeted towards active researchers.

## Methods

### Study setting and intervention description

Developing Excellence in Leadership, Training and Science (DELTAS) Africa, Phase One (2015–2021), was implemented by the Alliance for Accelerating Excellence in Science in Africa (AESA) with support from the Wellcome Trust and the UK’s Foreign, Commonwealth and Development Office (FCDO). DELTAS Africa funded 11 African-led research consortia to implement cutting edge collaborative research and training programmes spanning 54 institutions from across the continent. The DELTAS Africa CPE seed fund was designed to strengthen CPE capacity of DELTAS Africa early career researchers, and consortia staff more broadly, as well as pilot programmes of activity to promote societal impact of DELTAS Africa research. The seed fund was based on a ‘learn by doing’ model, with awardees expected to design and implement a CPE project aligned to a specified focal area. Originally planned to be administered across two rounds, Round One (August 2019 – March 2020) focused on CPE capacity strengthening; and Round Two (October 2019 – May 2020) focused on gender equity in research using innovative CPE initiatives. However, with the onset of the global COVID-19 pandemic a ‘Round Three’ (August – October 2020) was added with a focus on addressing the COVID-19 ‘info-demic’. The COVID-19 pandemic also severely disrupted the Round Two CPE projects which were in progress at that time. Accordingly, all Round Two CPE seed fund awardees were granted a no-cost extension until February 2021 and were supported to re-strategize incorporating remote engagement. 

The CPE seed fund was a competitive award funded by Wellcome and FCDO and administered by the African Academy of Sciences through AESA. Rounds One and Two were open to all PhD and Post-doctoral trainees affiliated to the 11 DELTAS Africa consortia. Round Three was further opened to specialist project management and science communications teams employed across the DELTAS consortia. Applicants were required to propose a CPE-project aligned with the respective focal area (capacity strengthening; gender equity; COVID-19 ‘info-demic’). A proposal template was provided to ensure consistency across applications. A total of 122 applications were received across the three funding rounds, with 13 awards conferred in Round One, seven awards in Round Two and five awards in Round Three. Each awardee received up to 25,000 USD (Rounds One and Two) or 35,000 USD (Round Three) to implement their respective project. The CPE projects were expected to be completed alongside the awardees existing consortia commitments and the awardees were expected to lead on all aspects of project implementation, including financial management and monitoring and evaluation (M&E). Round One and Two awardees were invited to a three-day induction meeting prior to implementing their respective project (this meeting was not held for Round Three awardees due to COVID-19 related travel restrictions in place at that time). The induction meeting included introductory lectures on CPE, project design and M&E. Each awardee was also provided personalised support during this meeting to refine their proposed CPE projects as needed. A limited amount of specialist CPE and M&E support was available to awardees during the project implementation period, primarily accessed in the form of remote telephone support on an ‘as needed’ basis. Awardees could also access a wide range of research support services through their respective DELTAS consortia. A summary of all 25 awards is included in the Extended data. 

### Data collection

This paper presents findings obtained from a mixed-methods process and performance evaluation of the DELTAS CPE seed fund independently conducted by the Centre for Capacity Research, Liverpool School of Tropical Medicine, UK, in partnership with the African Population and Health Research Center, Kenya. The evaluation drew on three data sources described in turn below.

A knowledge, attitude and practice (KAP) survey was administered to the 25 CPE project awardees at two time points: 1) prior to project commencement (i.e. pre-survey); and 2) following the submission of their respective ‘end of project’ reports (i.e. post-survey). The surveys were designed to measure changes in awardees CPE attitudes, knowledge, and proficiency as well as their experiences of project implementation and of the various support provided to assist them both prior to and during project implementation. Most questions included in the survey were developed by the research team and pilot-tested prior to use, although a small number of questions included in the post-survey were adapted from a previous survey of factors affecting public engagement by researchers conducted in the UK ^8^. These included: ‘How well equipped do you feel to engage with the public on your research or subject?’; and ‘How likely is it that you will engage with another CPE activity in the next 12-months?’. Follow-up questions exploring barriers and enablers to CPE participation were also adapted from the UK survey. All surveys were administered online via the ‘ Online Surveys’ platform. Participation was both anonymous and voluntary. An information sheet was included with the initial survey invitation, which was sent via email with a link to the survey form. Two ‘reminder’ messages were sent, also via email. Each survey was ‘live’ for a four-week period. In total, 23/25 awardees completed the pre-survey, and 22/25 awardees completed the post-survey. The pre- and post-survey questions can be found in the Extended data. 

Semi-structured interviews were completed with a sub-sample of awardees at the completion of their respective projects and with an AESA focal person towards the end of the grant period. Participation was voluntary, with an invitation extended to all 25 CPE seed fund awardees and to a small number of administrators and stakeholders involved in the CPE scheme and nominated by AESA. All interviews were approximately 60-minutes in length and completed remotely by the programme evaluation team and followed a structured interview guide. The interview guide explored: changes in CPE knowledge, attitudes and experience; experiences of the CPE award application, project preparation, implementation and outcomes; project monitoring, evaluation and reporting; and ‘other’ comments as deemed important by the interviewee. A total of nine interviews were completed: eight with CPE awardees and one with a programme administrator. Of the eight awardees interviewed, four were Round One, one from Round Two and three from Round Three. Five of the eight were PhD/post-doctoral fellows and three were research support staff. The interview guide can be found in the Extended data.

All 25 CPE seed fund awardees were required to submit a written ‘end of project’ report at the conclusion of their respective projects. A report template was provided which consisted primarily of narrative fields including: a description of the project aims; a description of achievements made against planned activities; a description of any delays faced and how these were mitigated; an outline of achievements against a project-specific M&E framework; a description of any challenges faced and lessons learnt; a description of dissemination activities; specific examples of value for money; an overview of high-level risks and how they were managed; and any additional feedback. All 25 awardees completed this report.

### Data analysis

Quantitative survey data were imported from the online surveys platform into a STATA (version 14) database for analysis. The Mann-Whitney U test was used to compare between group differences in CPE attitude, knowledge, and proficiency ratings pre- and post-survey. All other quantitative analyses were limited to descriptive statistics only. 

All interviews were transcribed in full. Free text data from the KAP surveys was imported into an Excel file. Interview data and free text data obtained from the KAP survey were analysed using a framework method ^14^. The framework was informed by the interview guide. The transcripts and free text responses were independently coded and entered into the framework by two reviewers (LMw and JP). Discrepancies between reviewers were resolved through consensus agreement. Once completed, the recorded entries in the framework were then thematically organised. End-of-project report content was synthesised under common headings, in line with the final report structure. Extracts from the nine interviews are coded T001-T009, extracts from the 25 end-of-project reports are coded R001-R025 and extracts from the KAP survey are coded S001-S022 (note: extracts are only presented from the post-survey). All codes have been randomly assigned to ensure anonymity, although they are consistently applied (e.g., all extracts sharing the same code are from the same individual).

### Ethical considerations

Ethical clearance was obtained from AMREF Ethics and Scientific Review Committee, Kenya (AMREF-ESRC P819/2020) and Liverpool School of Tropical Medicine’s Research Ethics Committee, UK (LSTM REC 20-005). A research permit was issued by National Commission for Science, Technology, and Innovation, Kenya. All survey and interview participants provided written informed consent. The right to use the project reports – as with all project documentation – was provided by the implementing organisation. 

## Results

### Experience of the DELTAS Africa CPE seed fund

A strong theme to emerge from the qualitative data was just how ‘new’ all elements of the CPE award were to many of the awardees. As a task, CPE was new to most interviewees, but so too was the experience of preparing and leading a substantive project, inclusive of project management, financial management and M&E. This is well illustrated in the following excerpt which highlights both the inexperience of many awardees as well as the variety of tasks that they were responsible for:

*“So, this was my first major grant... So, I think that in itself was daunting. But then, also, because I just didn’t have experience with writing a grant for that kind of money, it was a little hard. [In addition] I sacrificed my weekends to run this play [a component of her CPE project] and the teaching and the training. And I first had to teach them about TB and teach them about immune cells and what they are and how they behave, so that they know how to personalise them. And then putting together the script. Directing. Recording. I was the cameraman as well. And then designing the logo for the CPE. And, wow, the paperwork. And getting the production. I was like, wow, procurement. I was procurement. I was finance. I was everything.”* **T001**

Assuming responsibility for so much without substantial prior experience was often somewhat overwhelming for interviewees. This could result in some aspects of project delivery not receiving as much attention as perhaps was required. For example, when asked to comment on the M&E reporting expectations, one interviewee noted:

*“I had a lot on my plate. It's not like the reporting was demanding. But because it was in the mix of all these other things that I had to deliver on, it became a mammoth task also to do.”* **T007**

The multi-dimensional demands of the CPE award were exacerbated in some instances by the awardees’ relative inexperience in leading complex projects and, in particular, not fully utilising available resources within their respective consortia to ease the burden. One interviewee, when asked why they did not utilise consortia resources to a greater extent, commented:

*“I think it’s a little bit of being naïve. And it’s also a little bit of just placing the entire project on my shoulders and not knowing that I can share the load, if I can say it that way. But I think because I was just so into this mindset of this is my project, I have to make it work, I didn’t really allow myself to get as much help as I could have or should have. And so, yes, and I think that’s why I didn’t engage with [consortium name] as much as I should have or probably could have, and with the AAS in terms of coordinating the project.”* **T009**

Compounding the range of new activities and responsibilities awardees had to come to grips with was the limited time available in which to complete their respective projects. Interviewees consistently stated the six-month implementation period was too short:

“ *The project implementation timelines were too tight. It limited the scope of activities and levels of engagement considering the initial challenges experienced in setting up the activities.*” **R005**

For many interviewees the CPE award was considered quite disruptive to their PhD progress or routine roles and responsibilities. This was not presented as a negative but as an acknowledgement of their experience and a reflection of how the award was implemented as a stand-alone project on top of an already busy workload:

*“It was very hard. I totally didn’t anticipate the workload. Totally. So it got me off guard considering that we already had our other planned activities. So totally got me off guard. Quite a lot of effort to be put in and a lot of hours.”* **T005**

Time-related challenges were also the most frequent response to the question ‘What was most difficult about implementing your proposed CPE project?’ included in the post-survey. Of the 22 awardees who provided a response, nine were related to (lack of) time. Other responses included: delays experienced with internal institutional bureaucracies, typically related to procurement or funds disbursement (n=5); external factors outside of the awardees control (n=4); practical challenges with engaging target audiences (n=4); COVID-19 related challenges (n=3); the acquisition of skills necessary for project implementation (n=2); and language-barriers (n=1). 

The range of challenges reported in the post-survey were similarly reflected in end-of-project reports. Internal processes such as gaining required ethical approvals or adhering to institutional procurement policies often took considerably longer than awardees had anticipated, this was especially the case when procuring novel items or services. Delays were often experienced due to external factors associated with engaging target communities. For example, many of the projects that sought to engage school students were delayed due to National regulations that would not allow school-based activities during national examination periods, nor was it practically possible to engage students in a school context during a holiday break. Similarly, projects that required the awardees to obtain permits from National ministries often resulted in delays or the modification of projects.

Despite these challenges, the CPE award experience was invariably described in positive terms and the implementation process was widely considered a great learning opportunity. Frequently reported learnings included the need to be highly adaptive in response to the inevitable challenges that arose, being flexible in response to community needs, and the importance of adequate funding. Illustrative quotes include:

*“The main lesson l learnt from these engagement activities was the need to have an open mind and flexible approach. Things will not always go as planned, but it is essential that one can quickly respond and adjust the plans accordingly.”* **R009**

*“Just like developing a vaccine needs time, public engagement needs adequate time. You are not just out to send a series of advertisements, but you are out to build public trust and make the public part of your journey. That is one process that calls for patience and strategy and of course lots of consultations from all relevant authorities and stakeholders.”* **R005**

### Changes in CPE attitudes, knowledge, and proficiency

**Figure 1.**
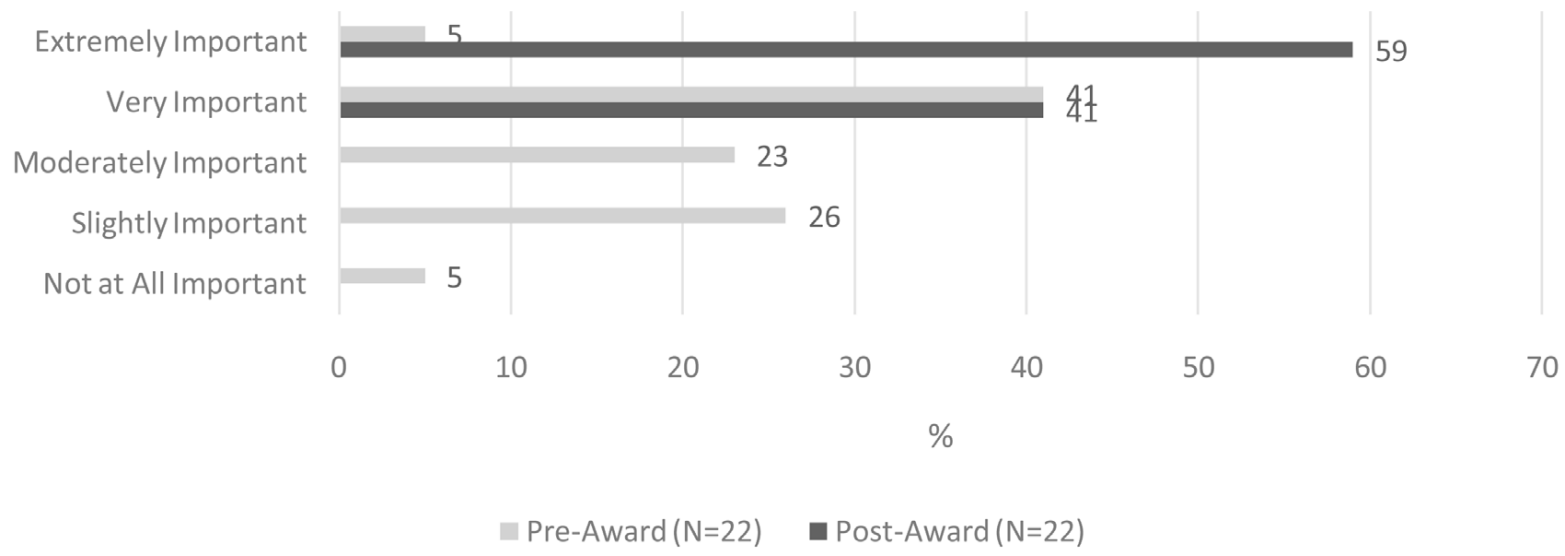
Perceived importance of CPE, pre- and post-CPE project implementation.

One hundred percent of awardees across all three funding rounds described CPE as either ‘very important’ (41%) or ‘extremely important’ (59%) following completion of their respective CPE projects ( Figure 1). This represents a 54-percentage point increase compared to baseline. The ‘between survey’ difference in perceived importance of CPE reached statistical significance (z= -4.613, p <0.001).

**Figure 2.**
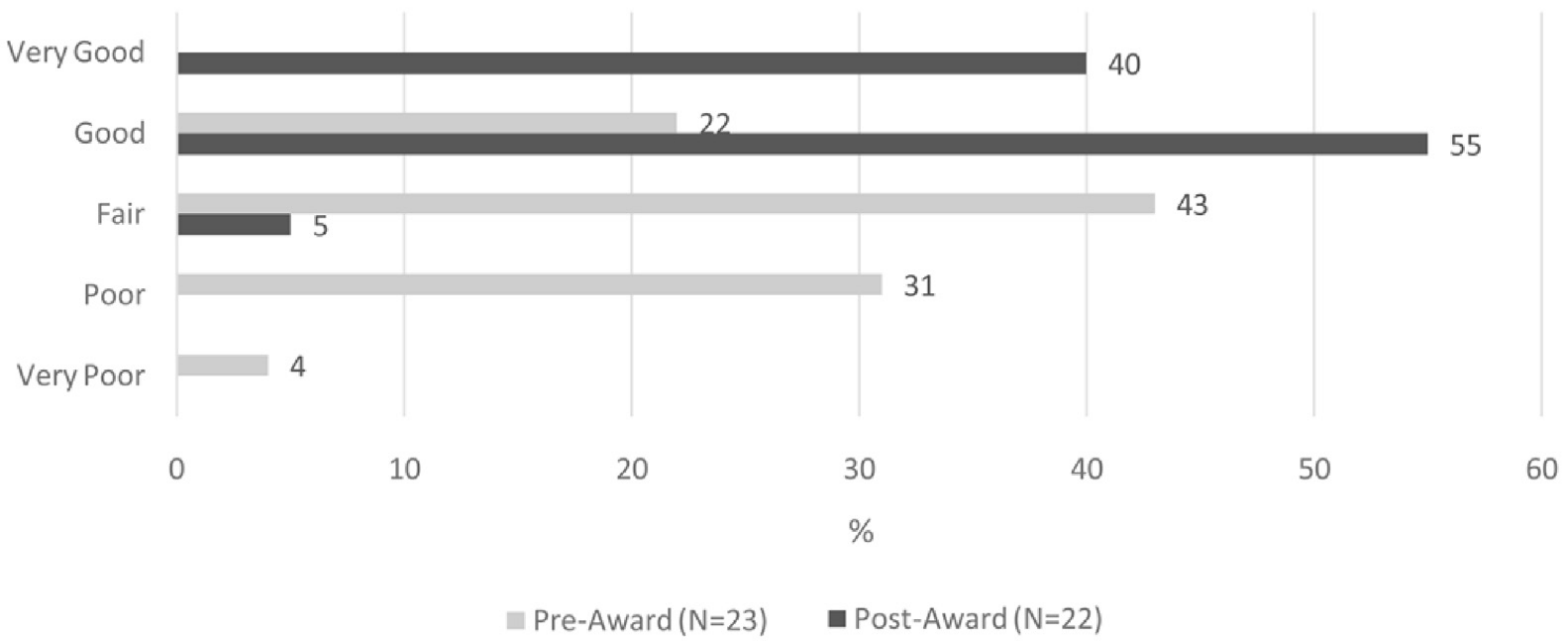
Self-rated CPE knowledge, pre- and post-CPE project implementation.

Ninety-five percent of awardees across all three funding rounds rated their CPE knowledge as either ‘good’ (55%) or ‘very good’ (40%) following the completion of their respective CPE projects ( Figure 2). This represents a 73-percentage point increase compared to baseline. The ‘between survey’ difference in self-rated CPE knowledge reached statistical significance (z= -5.042, p <0.001).

**Figure 3.**
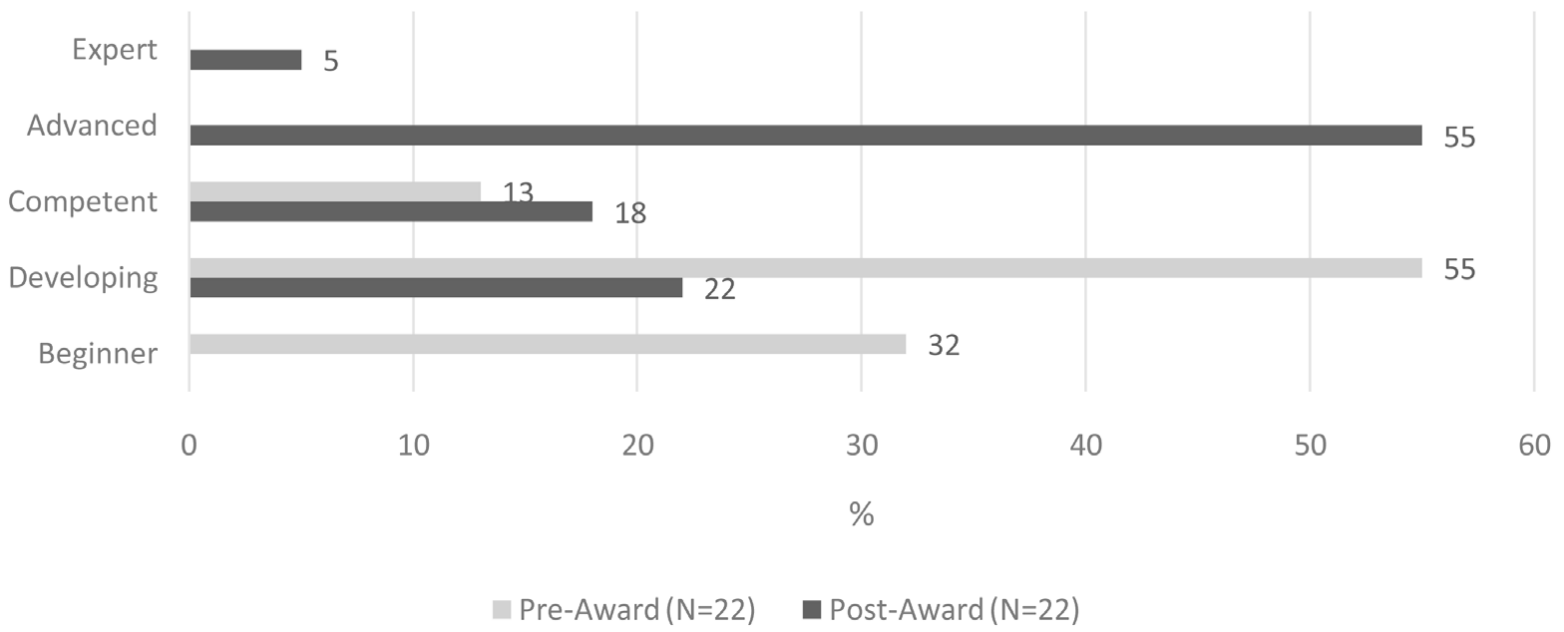
Self-rated CPE proficiency, pre- and post-CPE project implementation.

Sixty percent of awardees across all three funding rounds rated their CPE proficiency as either ‘advanced’ (55%) or ‘expert’ (5%) following completion of their respective CPE projects ( Figure 3). This represents a 60-percentage point increase compared to baseline. The ‘between survey’ difference in self-rated CPE proficiency reached statistical significance (z= -4.688, p <0.001).

**Figure 4.**
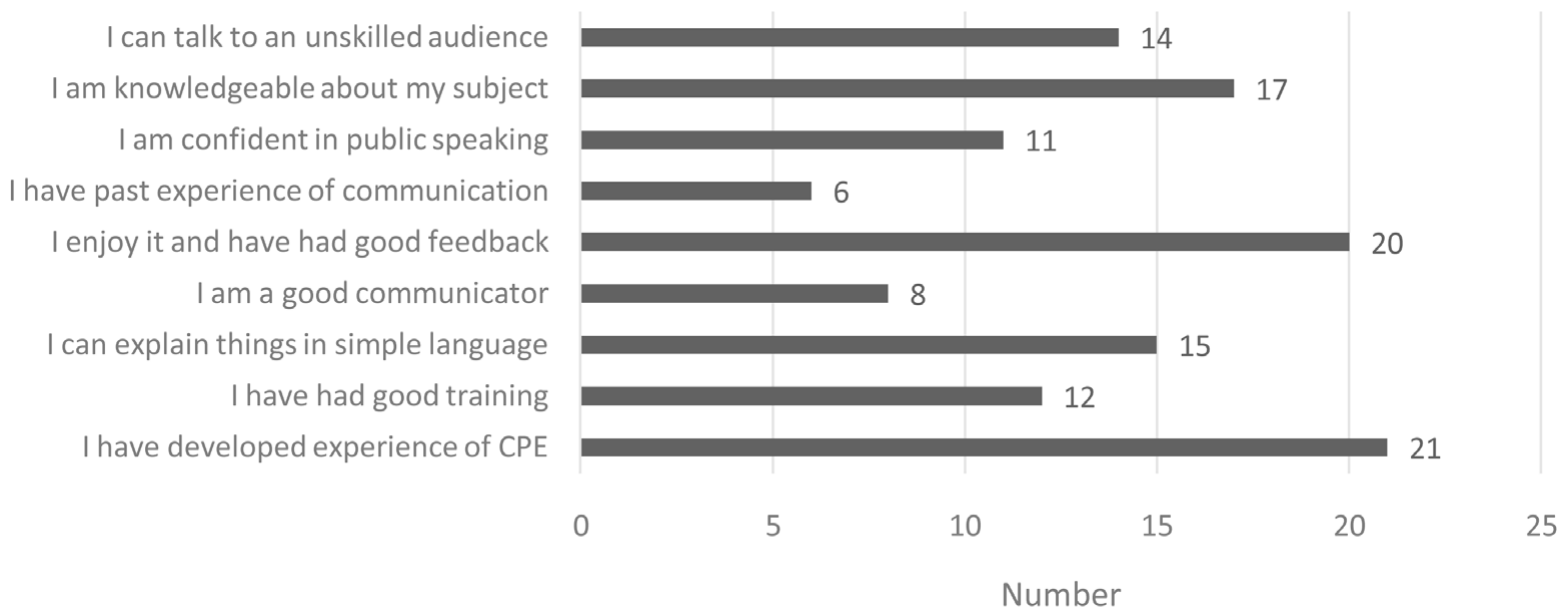
Reported reasons for feeling either 'very' or 'fairly' well equipped to engage with the public.

Post-survey participants were asked ‘How well equipped do you feel to engage with the public on your research or subject?’, in response to which 68% reported ‘very well equipped’ and 32% ‘fairly well equipped’. Participants were then asked to select from a list of nine possible response options ( Figure 4), the ‘reasons which best describe why you feel 'very' or 'fairly' well equipped to engage with the public?’. Participants could select multiple response options. As shown in Figure 4, the most frequently reported reasons included: ‘I have developed experience of CPE’ (21/22); ‘I enjoy it and have had good feedback’ (20/22); and ‘I am knowledgeable about my subject’ (17/22).

The qualitative data closely aligns with the KAP survey results. Six of the eight awardees interviewed reported having little or no prior knowledge or experience of CPE and attitudes were often indifferent as evident in the following comment:

*“I had treated community and public engagement as an additive, like it's an afterthought when you're doing research.”* **T007**

Where attitudes towards CPE were somewhat apathetic, and indeed even amongst those with more supportive attitudes, the motivation to apply for the award partly stemmed from the career advancement opportunities the CPE seed fund award represented: it was competitive, the available funds were relatively large for an early career award and successful applicants would gain valuable ‘principal investigator’ experience. Nevertheless, all interviewees reported a marked and often profound shift in their respective CPE attitudes, knowledge, and proficiency because of the seed fund experience. For example:

*“I started as someone who basically knew nothing about how to do CPE. Since that [CPE award experience], I think I’ve really grown in leaps and bounds in terms of understanding engagement. And I now consider science engagement as a possible career path for me.”* **T009**

### CPE practice and intentions post-award

**Figure 5.**
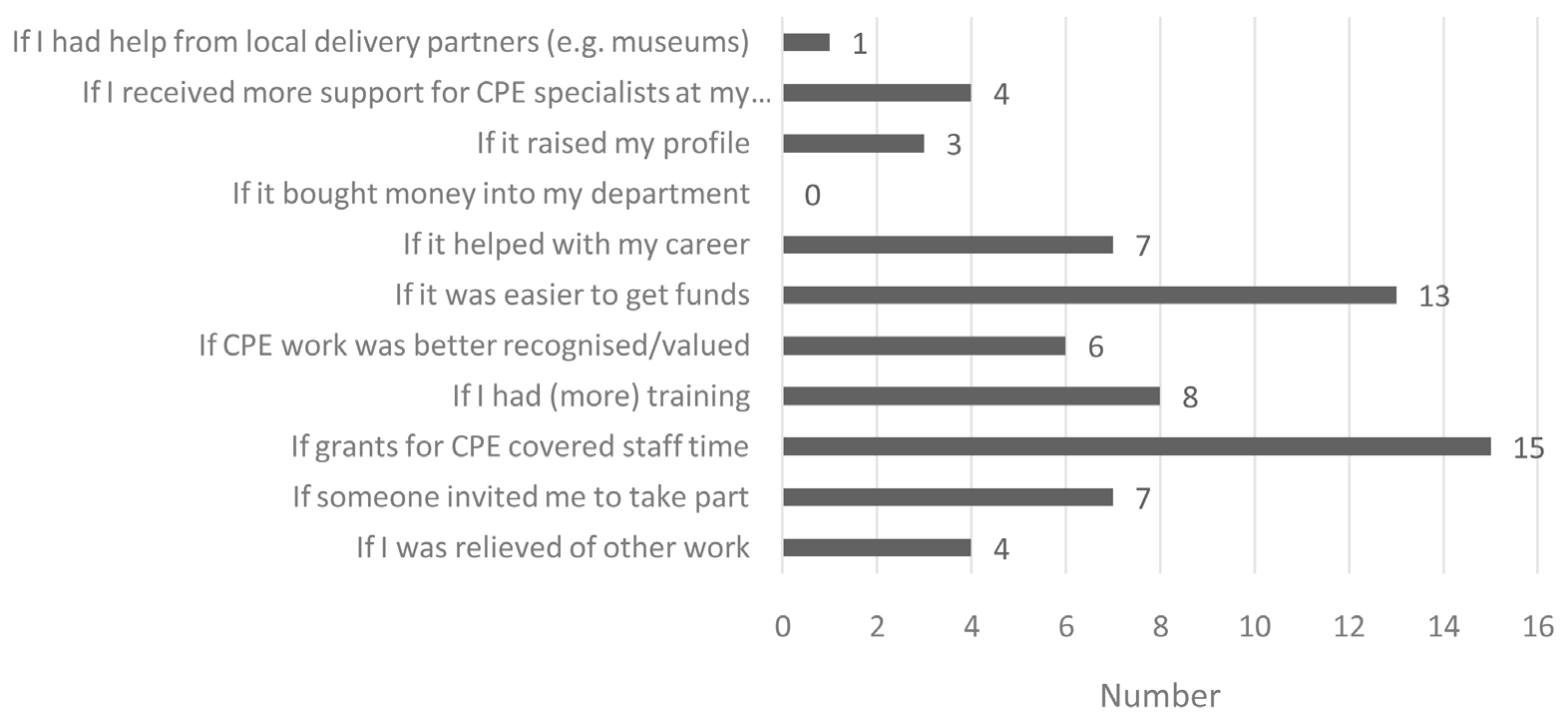
Reported factors that would encourage awardees to get more involved in CPE (N=22).

Commitment to continued CPE activity post-award was evident in the KAP survey data. Post-survey participants were asked ‘How likely is it that you will engage with another CPE activity in the next 12-months?’, in response to which 59% reported ‘definitely will’ and 41% ‘probably will’. Participants were then asked to select from a list of 11 possible response options ( Figure 5), the ‘three main factors that would encourage you to get more involved in CPE?’ and participants could select multiple response options. As shown in Figure 5, the most frequently reported factors included: ‘If grants for CPE covered staff time’ (15/22); ‘If it was easier to get funds’ (13/22); and ‘If I had (more) training’ (8/22).

As evidence of this commitment, a number of interviewees were still engaged in activities related to their CPE projects post-award or were seeking opportunities to further utilise project outputs. 

*“We’re still in the process of trying to think about where else we can put the art [CPE project output] to continue the dialogue and discussion around COVID-19 with the community but also around promoting this kind of engagement project with others. So I quite like that aspect that the art isn’t just… I feel the art is still the project continuing.”* **T004**

Equally without exception, all interviewees were adamant that they would integrate CPE within their respective research work going forward:

*“I would actually do this again. And what it means is that in future projects, whether a post-doc project or a full-time research project, I would include CPE both in a timeline of the implementation as well as in the budget line of the implementation. So it then becomes an integral part of the project and it doesn’t disrupt.”* **T003**

Many interviewees were also either actively engaging in new CPE activities or seeking funding to allow them to engage in new activities:

*“So, based on this project [the CPE project], what we are doing now is to replicate or implement this kind of activities in the countries where our fellows are working…before the annual [DELTAS consortium] meeting I present all the plan and we agree. The [management] give the okay for that…And now it is done, so we will carry out these activities very soon.”* **T002**

One interviewee had even been successful in obtaining funding to start a new research project in a subject area informed by her CPE activities:

Interviewee: *“I now have this additional interest to just, stemming from the stories that the young people shared, I felt there was a need to look into that. So, I was just recently awarded another grant from [consortium name], where I will be working with [research institution] to introduce self-testing for HIV and STIs among young people in Botswana. We just submitted to our IRB now. And as soon as we get approval, I’ll be heading to Botswana for that project.”*

Interviewer: *“And you see a direct link between your CPE involvement and that piece of research work?”*

Interviewee: *“Definitely. I would have never thought of doing that otherwise.”* **T001**

## Discussion

The study findings suggest the DELTAS Africa CPE seed fund functioned well as a capacity strengthening initiative. The combined survey, interview and end of project report data all uniformly revealed improvement in awardees’ self-reported CPE knowledge and proficiency by completion of their respective projects. The CPE knowledge and experience of most awardees prior to commencing their respective seed fund projects was extremely limited. This finding is consistent with previous studies among early career researchers ^8^ and further reinforces the need for initiatives such as the CPE seed fund. However, this finding also suggests we must be cautious not to overstate the extent of CPE capacity improvement among the study cohort. Whilst the self-reported data were highly indicative of substantial capacity gains, it was not possible to objectively assess the extent of any such gain and many (if not all) of the awardees would still likely benefit from additional CPE training and experience. It is perhaps with this in mind that the apparent shift in attitudes supportive of CPE also reported by awardees was especially encouraging. This attitudinal shift is likely to motivate many of the awardees to continue to engage in CPE activities post-award, with already some evidence for this, leading to further capacity gain. In this sense, the seed fund appears to not only have facilitated CPE capacity strengthening during the life course of the award, but it has also served as a launching pad for continued CPE practice and professional development. Given internal motivation is considered one of the more influential drivers of sustained behaviour change ^15^, it is this attitudinal shift that may prove most central to continued CPE practice among the DELTAS Africa CPE seed fund cohort.

All awardees described their seed fund experience in positive terms, despite invariably finding it more challenging than originally anticipated, suggesting that ‘learn by doing’ was not only an effective CPE capacity strengthening/awareness raising approach, but also highly acceptable among the target cohort. In fact, most of the learning experiences reported by awardees stemmed from challenges encountered through the process of implementing their project in practice. Confronting and resolving challenges is an intended learning mechanism in ‘learn by doing’ approaches and, as evident here, can engender feelings of satisfaction and accomplishment among participants ^16^. Thus, the ‘learn by doing’ approach employed in the CPE seed fund appears to have functioned in a manner consistent with the underlying intent. Nevertheless, the study highlighted common issues that compounded the various project-specific challenges faced which could potentially be addressed in future versions of the scheme (or application of similar schemes by other organisations). The limited time available to implement the CPE award, and the structure of the seed fund as a stand-alone activity were two such compounding issues. Allowing more time for implementation and more closely integrating the CPE projects (Round One and Two only) with the awardees’ respective research projects from the outset could potentially have reduced stress and maximised outcomes. Awardees would also have benefitted from more wide-ranging project management training and support, especially in the early stages of project planning and preparation. Importantly, if future versions of the scheme were to integrate the award more closely with an underlying research project, then resources and supports should still be protected specifically for CPE and outcomes reported independently of the wider study findings. These actions would ensure the CPE component is not diluted or neglected in the face of potentially competing project demands.

The grant-based structure of the CPE seed fund appealed to the awardees. The structure and application process mimicked that of a typical research grant call: the call was competitive, applicants were required to submit a project proposal, it was for a reasonably substantial sum of money and success conferred principal investigator status. These features of the award speak to a widely understood pathway of scientific career progression; namely, obtaining grant income and principal investigator experience ^17^. Obtaining a track record in these areas was especially desirable to early career researchers and, for most, the award was a first experience of a successful grant application. The appeal of the CPE seed fund in this structure presents as an important consideration given the somewhat ambivalent attitudes many awardees reported with respect to CPE pre-award. The motivation to apply was often as much about career building as it was gaining CPE experience. This suggests the seed fund was partly successful because it was able to leverage off a recognised means of scientific career progression to advance a CPE capacity strengthening agenda. The grant-based structure, combined with the intensive workload the ‘learn by doing’ project-based approach necessitated, are perhaps most well suited to an early career researcher cohort. Evidence indicates CPE capacity strengthening is also required among mid-to-senior career researchers ^8^. However, more established researchers may not have sufficient time to be as ‘hands on’ as their early career counterparts, thereby negating the benefits of the ‘learn by doing’ approach and are perhaps less in need of the grant income and principal investigator experience. Therefore, a diverse array of CPE capacity strengthening initiatives may be needed, variously targeting researchers at different stages of career progression.

This study was not without limitation. The CPE seed fund was a pilot programme with the total number of awardees was limited to 25. Thus, the study data pertain to a small sample of African researchers/research professionals all of whom belonged to a relatively well-funded research capacity strengthening network (DELTAS Africa). As such, the findings may not be readily generalisable beyond the study setting, especially for contexts in which early career researchers do not have access to effective research support. The study findings suggest that provision of the latter would be essential to further implementation of the same or similar CPE seed fund award; implying that effective research support would need to be sourced elsewhere if not readily available to future awardees in their home institution. Changes in CPE knowledge, attitude and proficiency were largely measured by participant self-report. The consistency across data sources provides some reassurance that most, if not all, awardees improved on some or all of these three dimensions; however, it is not possible to determine the extent of any such improvement in the absence of more objective measurement. Participants may also have been reluctant to express negative or critical opinions of the CPE seed fund given it was delivered within the frame of the same initiative in which they were employed or supported to complete their post-graduate studies (DELTAS Africa). To reduce the possibility of this form of bias, all surveys were anonymous, issues of anonymity and confidentiality were addressed in the informed consent process prior to interview and all data were collected by researchers independent of both the DELTAS Africa initiative and the CPE seed fund. 

## Conclusion

The DELTAS Africa CPE seed fund appeared to work successfully as a CPE capacity strengthening platform and as a vehicle for fostering interest in CPE longer-term. Any future or similar versions of this scheme could consider some modifications in terms of duration, timing of implementation and the types of support provided. Recipients are also likely to require continued CPE training and support post-award to further their professional development and different forms of capacity strengthening intervention may be required for researchers at different career stages.

## Data availability

### Underlying data

This paper drew on three data sources including survey data, interview transcripts, and end of project reports. 

The survey dataset is publicly accessible:

Harvard Dataverse: DELTAS CPE Seed Fund Evaluation – Dataset and supplementary information. https://doi.org/10.7910/DVN/OVN8DK ^18^

This project contains the following underlying data:

- DELTAS CPE Seed Fund Evaluation_KAP survey dataset (a copy of the KAP survey dataset)

Data are available under the terms of the Creative Commons Attribution 4.0 International license (CC-BY 4.0).

De-identified interview transcripts and end of project reports are available from the research group on request for the purpose of informing further research and on the condition that they will not be published in part or in entirety. Requests for access to this data can be made to the research group at the Centre for Capacity Research, ccr@lstmed.ac.uk, or to the LSTM Research Ethics Committee, rec@lstmed.ac.uk.

### Extended data

Harvard Dataverse:DELTAS CPE Seed Fund Evaluation – Dataset and supplementary information. https://doi.org/10.7910/DVN/OVN8DK ^18^

This project contains the following extended data:

- DELTAS CPE Seed Fund Evaluation_Summary of awarded projects (a summary of the 25 CPE projects funded through the award)
- DELTAS CPE Seed Fund Evaluation_KAP survey pre (a copy of the pre-project questionnaire)
- DELTAS CPE Seed Fund Evaluation_KAP survey post (a copy of the post-project questionnaire)
- DELTAS CPE Seed Fund Evaluation_Interview guide (a copy of the interview guide)

Data are available under the terms of the Creative Commons Attribution 4.0 International license (CC-BY 4.0).
