# Supplementary material for: Strengthening capacity for community and public engagement (CPE): a mixed-methods evaluation of the ‘DELTAS Africa CPE seed fund’ pilot
Source: Wellcome Open Res. 2022 Mar 17;7:96. [Version 1] doi: 10.12688/wellcomeopenres.17665.1 (PMC9386295; doi:10.12688/wellcomeopenres.17665.1)
Supplement: Peer review history

# Peer review: Reviewer response for version 1

**DOI:** 10.21956/wellcomeopenres.19542.r51597

**Published:** 2022-08-17

**Authors:** Catherine van de Ruit

**Affiliations:** 1 Health and Society, Health and Exercise Physiology, Ursinus College, Collegeville, PA, USA

The authors have identified a vital area of inquiry – to strengthen community engagement in academic research (CPE). The evaluation of the DELTAS project offers insight into ways to mentor and train researchers to undertake CPE. It would be great if the article could go further in substantive description of how the DELTAS trains and mentors researchers in CPE and provide  more depth about concrete knowledge and tools the researchers gained during the process.

 Detailed suggestions

 Abstract and Introduction

- Abstract introduces how the DELTAS Africa CPE seed fund’ involves a “Learning by doing” approach – perhaps in the introduction you might explain what this entails? For example, what kinds of knowledge and tools were provided at every stage of the research process?
- While I understand word/page limits might be an issue perhaps in the introduction you might contextualize the CPE more. What kind of mentoring did grantees receive, what other trainings did grantees receive?
- Methods
  - Discussion of analytic rigor: data credibility and trustworthiness would be useful.
  - Perhaps you might add a summary table of participants projects and the kind of CPE they undertook– while this information is provided in the extended data perhaps you might introduce a short summary table where you categorize the projects such as school programs versus community initiatives. The research also spans infectious versus chronic conditions so you could also consider grouping studies into these different kinds of buckets. Region – you might condense the categories there by subregion for instance eastern, southern etc. This information would enable readers to gain a sense of the substantive content of the projects without having to go into extended data unless they want more detail of the different projects.
  - The article mentions that participants initially receive 3 days training and then follow up mentorship – perhaps say more about the content of the trainings and mentorship. Specifically what content on CPE is shared with participants so the reader gains more of a perspective of how DELTA’s approached CPE.
  - On page 4 the article says “The interview guide explored: changes in CPE knowledge, attitudes and experience; experiences of the CPE award application, project preparation, implementation and outcomes; project monitoring, evaluation and reporting.” However the results section does not really address the details of what kinds of CPE knowledge was gained and experiences of CPE – more information about this in the results section would contribute to improving understanding about how to mentor researchers on CPE

Results

- At the top of page 6 one of the interviewees mentions the importance of building public trust. Wonder if you might expand on this analysis more – was this the only person who mentioned public trust? If not please develop this idea more as this speaks to the kinds of changes in research practices needed to undertake robust publicly engaged research.
- Page 6-8 is the heart of your analysis discussing Changes in CPE attitudes, knowledge, and proficiency.  Can you give examples of what people said to make it more concrete. In the interview guide participants are asked to define CPE, explain how they used it in their projects and significant outcomes – it would be interesting to know more about their discussions about these topics.

Discussion

- Would it be possible to discuss what mentoring or public engagement strategies proved most effective as a way to advance knowledge of publicly engaged scholarship?

Is the work clearly and accurately presented and does it cite the current literature?

Yes

If applicable, is the statistical analysis and its interpretation appropriate?

Yes

Are all the source data underlying the results available to ensure full reproducibility?

Yes

Is the study design appropriate and is the work technically sound?

Yes

Are the conclusions drawn adequately supported by the results?

Partly

Are sufficient details of methods and analysis provided to allow replication by others?

Partly

Reviewer Expertise:

Global health, qualitative methods

I confirm that I have read this submission and believe that I have an appropriate level of expertise to confirm that it is of an acceptable scientific standard, however I have significant reservations, as outlined above.

# Peer review: Reviewer response for version 1

**DOI:** 10.21956/wellcomeopenres.19542.r51593

**Published:** 2022-08-15

**Authors:** Gunilla Bjorling

**Affiliations:** 1 Department of Neurobiology, Care Sciences and Society, Karolinska Institutet, Stockholm, Sweden; 2 Jönköping University, Jönköping, Sweden; 3 Kilimanjaro Christian Medical University College, Moshi, Tanzania

Thank you for a very interesting manuscript.

 It is well designed and an important study. However, the mixed-methods approach require a more specified interpretation of the results. In the method section the qualitative data analysis is not described enough. i.e. you should include a table over the coding, category and theme- process. The code cannot be a number. The category should sate the content in the text. It appears that it is not analysed enough. The themes are not clear. Please, describe it better, so it is easier to follow. The method gives implications on the results, which should be better organized. My suggestion is that you start with the survey and the open comments and describe that. After that you start with the qualitative section and describe the results under headings which are the same as the themes and subheadings, the same as the categories. 

 Furthermore, the study and the organisation of results could benefit from a statement of research questions in the method section. It is a very good project and the study is well described, but it need some improvements.

 Thank you!

Is the work clearly and accurately presented and does it cite the current literature?

Yes

If applicable, is the statistical analysis and its interpretation appropriate?

Partly

Are all the source data underlying the results available to ensure full reproducibility?

Partly

Is the study design appropriate and is the work technically sound?

Partly

Are the conclusions drawn adequately supported by the results?

Yes

Are sufficient details of methods and analysis provided to allow replication by others?

No

Reviewer Expertise:

Medical education, research methodology, nursing research

I confirm that I have read this submission and believe that I have an appropriate level of expertise to confirm that it is of an acceptable scientific standard, however I have significant reservations, as outlined above.

# Peer review: Reviewer response for version 1

**DOI:** 10.21956/wellcomeopenres.19542.r49273

**Published:** 2022-04-11

**Authors:** Mary Chambers

**Affiliations:** 1 Oxford University Clinical Research Unit, Ho Chi Minh City, Vietnam

This paper describes a novel, trans-African funding scheme to promote researcher-led community and public engagement. It offers important findings that support the call for more awareness of the need for PCE with research, and for capacity strengthening for African researchers to lead and conduct effective and appropriate engagement.

 The pilot scheme was set up as part of the Developing Excellence in Leadership, Training and Science (DELTAS) Africa Programme. PhD and post-doctoral researchers from any of the 11 African-led research consortia under the DELTAS Programme were eligible to apply for funding to conduct PCE projects alongside their research activities. This paper outlines the methods of setting up the seed funding scheme, how it was evaluated and the results of the evaluation. The authors anticipate that the evaluation findings will inform future iterations of the seed fund scheme as well extend current understanding of good practice in CPE capacity strengthening approaches targeted towards active researchers.

 The paper is well written, structured and the research process clearly presented. The evaluation of the PCE seed funding initiative is appropriate for the scale of the programme. The results are clearly presented in tables and in the text, and support the analysis and conclusions. I congratulate the authors for a well written and important publication, and their involvement in this scheme which will undoubtedly go a long way to strengthen engagement practise amongst African researchers. 

 My only suggestion for edits are:

- The Abstract doesn't present the extent of the challenges that are presented in the Results section.
    
- More specific ideas for modifications or improvements on a future scheme could be included in the Discussion/Conclusion. 

Is the work clearly and accurately presented and does it cite the current literature?

Yes

If applicable, is the statistical analysis and its interpretation appropriate?

Yes

Are all the source data underlying the results available to ensure full reproducibility?

Yes

Is the study design appropriate and is the work technically sound?

Yes

Are the conclusions drawn adequately supported by the results?

Yes

Are sufficient details of methods and analysis provided to allow replication by others?

Yes

Reviewer Expertise:

Public & Community Engagement

I confirm that I have read this submission and believe that I have an appropriate level of expertise to confirm that it is of an acceptable scientific standard.
